# The genetic relationship between systemic lupus erythematosus and risk of primary ovarian failure from a mendelian randomization study

**DOI:** 10.1038/s41598-024-59726-9

**Published:** 2024-04-24

**Authors:** Xiangfei Wang, Ruolin Mao, Meng Wang, Lixia Zhu, Lei Jin

**Affiliations:** Reproductive Medicine Center, Tongji Hospital, Tongji Medical College, Huazhong University of Science and Technology, 1095 Jiefang Ave, Wuhan, 430030 China

**Keywords:** Systemic lupus erythematosus, Primary ovarian failure, Two-sample mendelian randomization, Genetic association, Causality, Computational biology and bioinformatics, Genetics, Immunology, Rheumatology, Risk factors

## Abstract

Previous studies investigating the relationship between systemic lupus erythematosus (SLE) and primary ovarian failure (POF) generated conflicting results. To data, no mendelian randomization study has been applied to examine this association. In this study, genetic instruments for exposure (SLE) were selected from a GWAS study with 5201 cases and 9066 noncases. Outcome data for POF and three reproductive traits (age at menarche, age at menopause, and age at first live birth) were obtained from other eligible GWASs. To estimate causal association, the inverse-variance weighted (IVW) method (the main analyse), MR Egger test, weighted median, simple mode, and weighted mode were applied. Moreover, sensitivity analyses were conducted to ensure the robustness of the results. Estimated by the IVW method, SLE was suggested to be causally related to the risk of POF (OR = 1.166, 95% CI 1.055–1.289, *P* = 0.003) and delayed age at first live birth (OR = 1.006, 95% CI 1.002–1.010, *P* = 0.007), with no evidence of a causal association between SLE and age at menopause or menarche. The estimates were robust according to sensitivity analysis. In conclusion, the two-sample MR study supported a causal association between SLE and POF from a genetic aspect.

## Introduction

Systemic lupus erythematosus (SLE) is an aberrant condition characterized by excessive activity of the immune system, in which healthy cells and tissue can be mistakenly attacked. With loss of tolerance to autoantigens and an overresponse of immune cells, SLE manifests as a range of symptoms from mild fatigue and joint pain to severe damage to organs^1^. Although the symptoms of SLE can be controlled by medications, it is not curable^1^. The prevalence of SLE in North America was estimated to be 241/100,000 people and women of reproductive age were more commonly affected than men^2^.

Thus far, evidence has indicated that SLE might be associated with impaired ovarian function and female fertility. Specifically, primary ovarian failure (POF), as characterized by the traits of decreased sex steroid, premature menopause, and resistant ovary syndrome in women younger than 40 years old^3^, is found to occure more frequently in SLE patients than in healthy controls^4^. However, due to the necessary use of drugs like cyclophosphamide (CYC) to treat severe SLE, it has been suggested that the association between SLE and POF might be attributed to medication. While after eliminating the effect of CYC, it is still controversial whether SLE has an effect on menstrual cycle^5,6^. The correlation between SLE and other biomarkers that evaluate the ovarian function and female fertility has also been investigated. It turns out that both ovarian reserve (evaluated by anti-Mullerian hormone (AMH)^7^) and pregnancy outcomes might be affected by SLE activity^8^. Nevertheless, in consideration of confounders such as the different doses of drug use as well as disease flare, it is difficult to provide concrete evidence about the association between SLE and female reproduction based on observational studies.

Mendelian randomization (MR) is applied as a method to investigate the causal relationship between risk exposure and outcomes by using genetic variants as instrumental variables for exposure^9^. Since genetic variants are randomly inherited from parents, they are unlikely to be influenced by confounders introduced by environmental factors^10^. Hence, MR can mimic the design of randomized trials and verify whether an observational relationship between exposure and outcome is consistent with a causal effect^11,12^.

In the present study, two-sample MR was therefore conducted to investigate the causal relationship between SLE and the risk of POF. Moreover, three other female reproductive traits (age at menarche, age at menopause, and age at first live birth) were selected as evaluated outcomes to further clarify how SLE impacts female reproduction. Our study provides evidence to achieve a better understanding of the relationship between SLE and female reproduction.

## Materials and methods

### Study design

**Figure 1:**
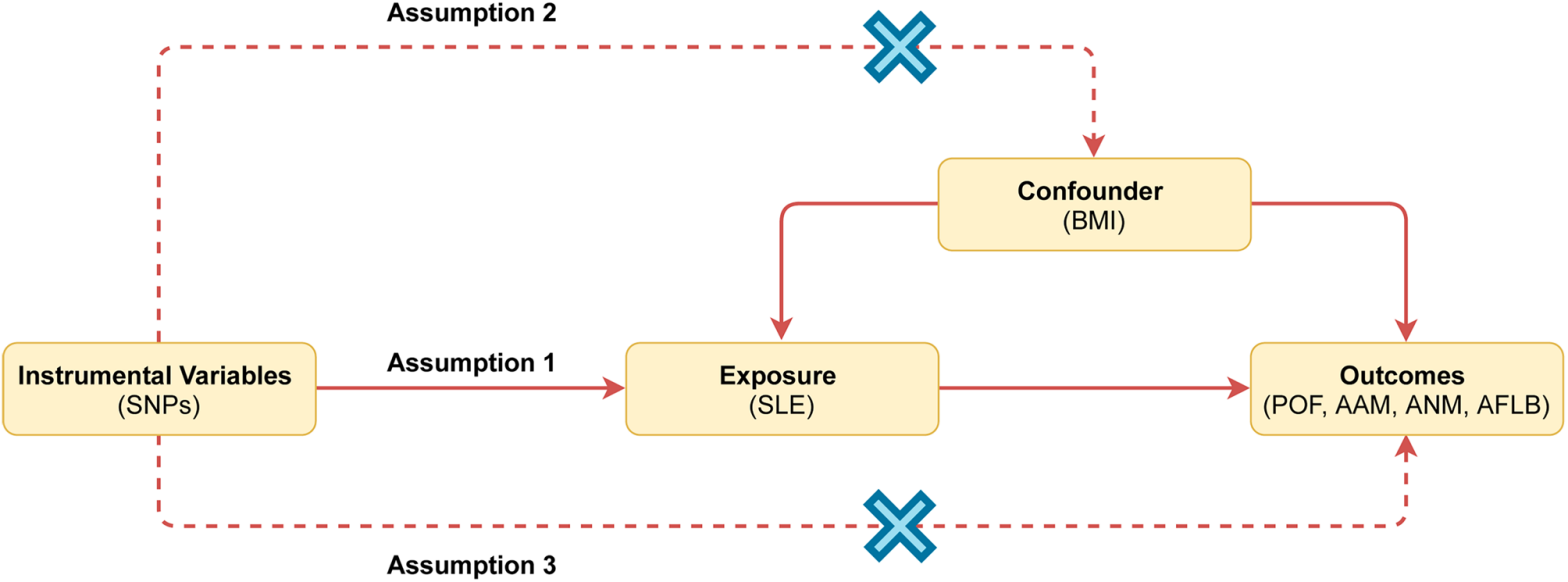
Assumptions of Mendelian Randomization studies. Three hypotheses are needed to obtain a persuasive conclusion from the two-sample MR. First, genetic instrument variables (SNPs) are directly associated with exposure (SLE). Second, IVs should be independent of confounders (BMI). Third, IVs affect the outcome (female reproductive traits) only through exposure (SLE). *Note* SNPs: single-nucleotide polymorphisms; SLE: systemic lupus erythematosus; BMI: body mass index; POF: primary ovarian failure; AAM: age at menarche; ANM: age at natural menopause; AFLB: age at first live birth.

**Figure 2:**
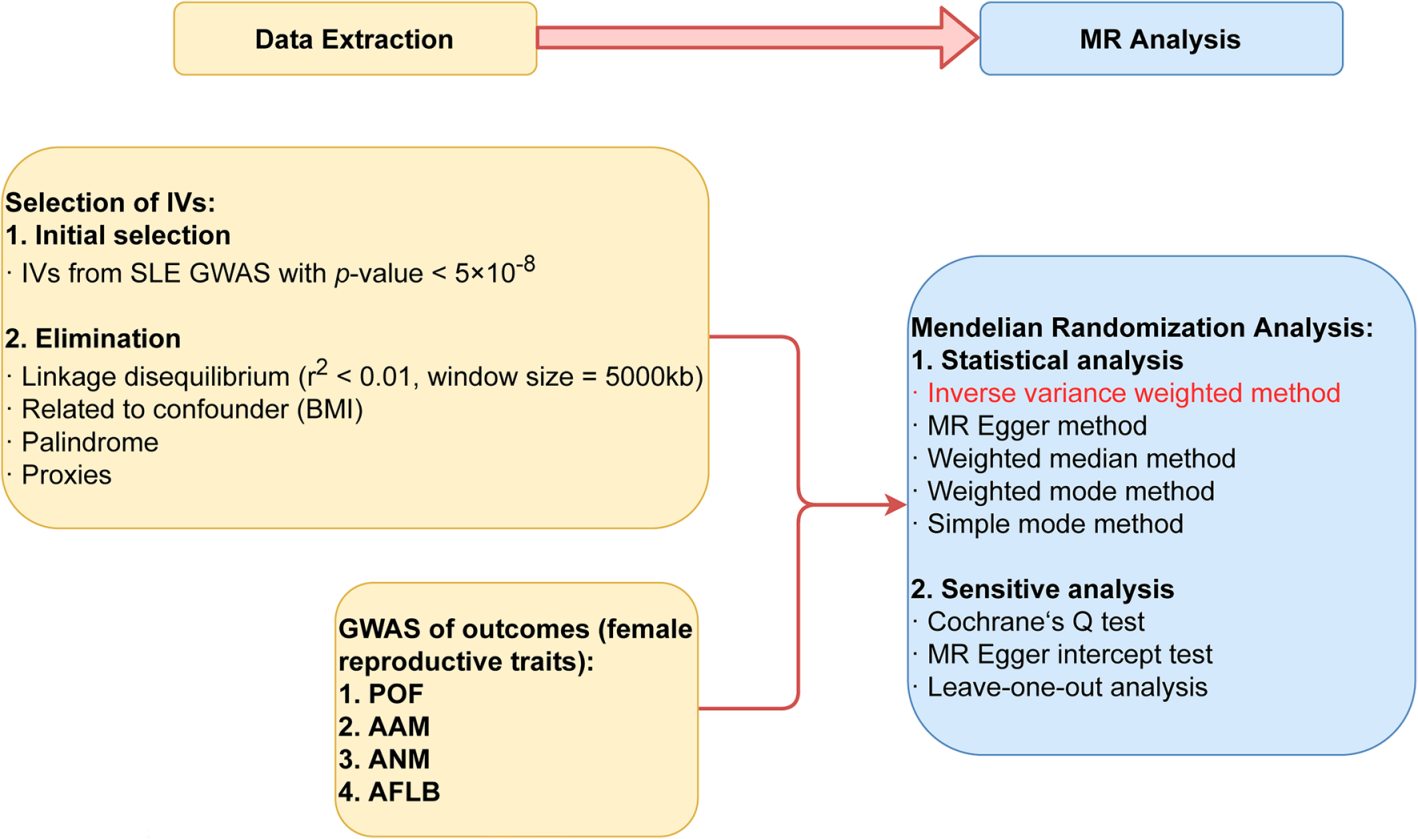
Overview of the present study design. *Note* MR: mendelian randomization; IVs: instrumental variables; GWAS: genome-wide association study; SLE: systemic lupus erythematosus; BMI: body mass index; POF: primary ovarian failure; AAM: age at menarche; ANM: age at natural menopause; AFLB: age at first live birth.

In this study, two-sample MR was performed to evaluate causal relationship between SLE and female reproductive traits. Three hypotheses need to be satisfied to obtain a persuasive conclusion from two-sample MR (Fig. 1). First, genetic instrument variables (IVs) are directly associated with exposure (SLE). Second, IVs should be independent of confounders (Body Mass Index (BMI)). Third, IVs affect the outcome (female reproductive traits) only through the exposure (SLE). An overview of the research design was demonstrated in Fig. 2. Ethical approval and informed consent were obtained in all of the studies included.

### Data source

The summary dataset for SLE (OMIM 152,700) was acquired from a large genome-wide association study (GWAS) conducted by Bentham et al.^13^. A new GWAS, a meta-analysis, and a replication study were involved, composing a sample size of 5201 cases and 9066 controls in total. As for female reproductive traits, the information of summary statistics was shown in Supplementary Table S1, including POF (254 cases and 118,482 controls) from the FinnGen study^14^, age at menarche (182,416 participants), age at natural menopause (69,360 participants), and age at first live birth (123,846 participants) from UK Biobank^15^. The data used for SLE and female reproductive traits were all restricted to European ancestry to avoid potential bias. This study was approved by the institutional review board (IRB) before initiation. The authors of the GWASs used for data collection in this study obtained their own ethical approval.

### Selection of SNPs

To conduct further MR analysis, IVs were selected from GWASs. For the exposure (SLE), SNPs with a *P* value lower than 5 × 10^–8^ were extracted as IVs from the corresponding GWAS. These SNPs were further pruned for linkage disequilibrium (*R*^2^ < 0.01 at a 5000-kb window) to ensure independency among SNPs. In addition, all of these SNPs were searched at PhennoScanner V2 to rule out SNPs associated with confounder (BMI) (*P* < 5 × 10^–8^)^16^. For outcomes (female reproductive traits), summary statistics were extracted from the GWASs, respectively. During the process, if selected SNPs were not present in outcome datasets, they were removed from further MR analysis instead of finding proxy variants. Palindromic SNPs with intermediate frequencies were also excluded. Furthermore, *F*-statistics were calculated using the proportion of variance (*R*^2^) developed from each SNP. The following equation was used: *F* = *R*^2^ × (N − 2)/[K × (1 − *R*^2^)] (*R*^2^ denotes the exposure variance explained by each IV; N is the number of samples in GWAS;and K represents the number of SNPs for analysis)^17^. If *F* > 10, we have reason to believe that the strength of IV is sufficient for further analysis without weak-tool bias.

### Statistical analysis

After harmonizing SNP statistics from SLE and female reproductive traits to ensure consistency, we conducted MR analysis using five methods. Among them, the main analytical method was the inverse variance weighting (IVW) method of different models, which assumes that all variants are valid and have balanced pleiotropy^18,19^. Other methods include the MR Egger test, weighted median, simple mode, and weighted mode. According to the slope of weighted regression of the SNPs-outcome association on the SNPs-exposure association, the MR Egger test gives a valid test of the null causal hypothesis and estimate of causal association even when all SNPs are invalid IVs^20^. Likewise, the weighted median method can provide an effect estimate even when invalid IVs were over 50%^21^. When the largest number of similar (identical in infinite samples) individual-instrument causal effect estimates is from valid instruments, the simple and weighted mode methods are consistent, even if most IVs are invalid^22^.

### Sensitive analysis

To perform a sensitivity analysis, we first used the intercept of the MR Egger test as an indicator to evaluate the directional horizontal pleiotropy^20^. Furthermore, the Cochrane Q test was conducted to estimate heterogeneity in effect sizes among IVs. And if the heterogeneity evaluated by the Cochrane Q test was high, the random model was applied. Otherwise, the fixed model was chosen. Third, a leave-one-out analysis was conducted to indicate that the causal relationship was not dominated by a single SNP. In addition, a funnel plot was constructed to demonstrate whether the pleiotropy existed.

### Ethical approval

The study was approved by the Ethical Committee of Tongji Hospital, Tongji Medicine College, Huazhong University of Science and Technology (TJ-IRB20211280). And GWASs used for data collection in this manuscript have obtained their own ethical approval respectively.

## Results

### Selection of instrumental variables

Fifty-nine IVs were extracted from the SLE GWAS. After searching each SNP in the PhenoScanner database, we excluded 3 SNPs related to BMI (rs204899, rs2736332, and rs3128929), a potential confounder. Subsequently, by harmonizing with 47 SNPs extracted from the outcome dataset, 46 SNPs were included in the following analysis. The final results of the screened SNPs were demonstrated in Supplementary Table S2–5. Among them, all *F*-statistics were above 10, showing no evidence of weak-tool bias.

### Causal association of SLE with POF

**Figure 3:**
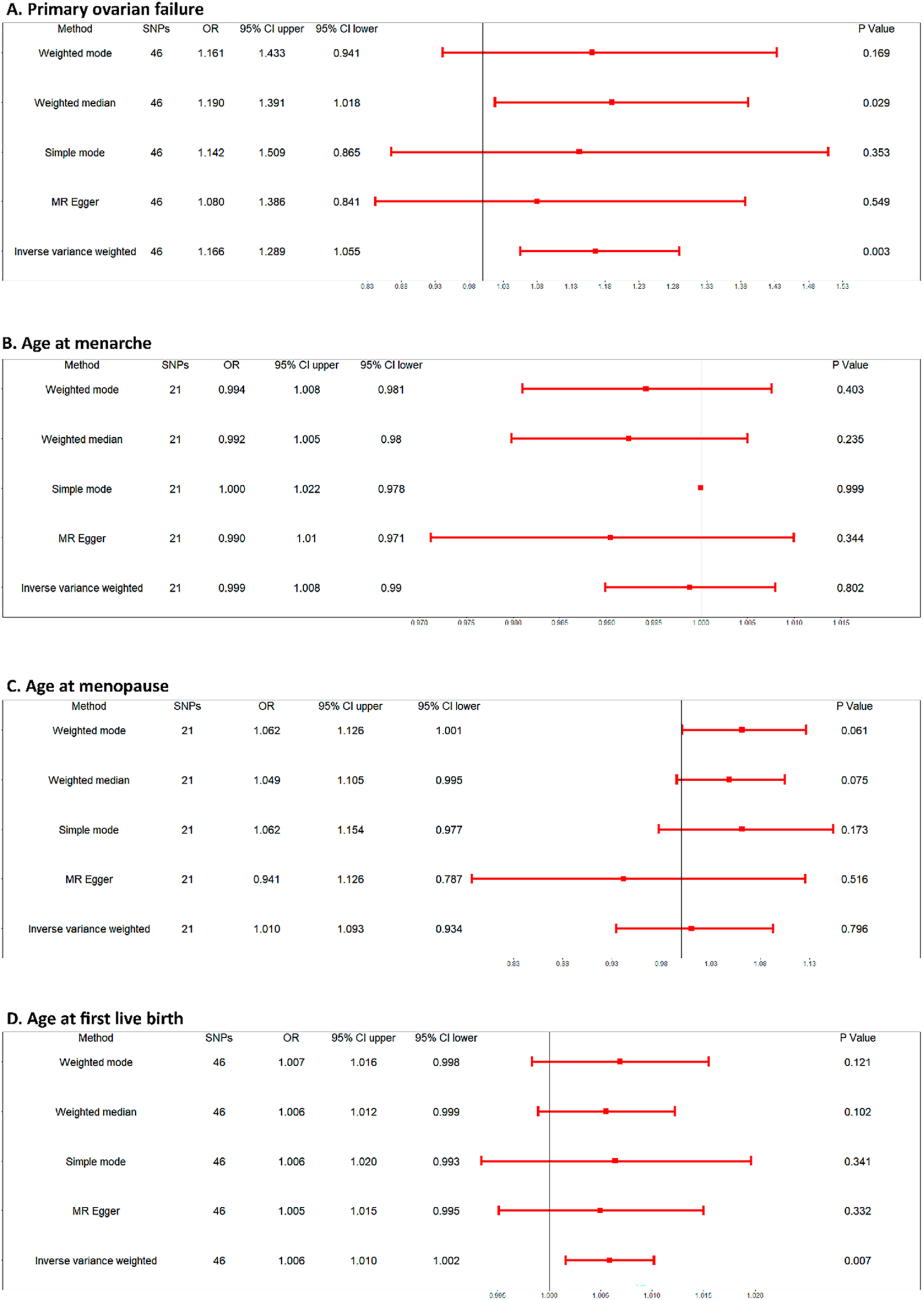
Forest plot of the association between SLE and reproductive traits by using five kinds of analysis method.

**Figure 4:**
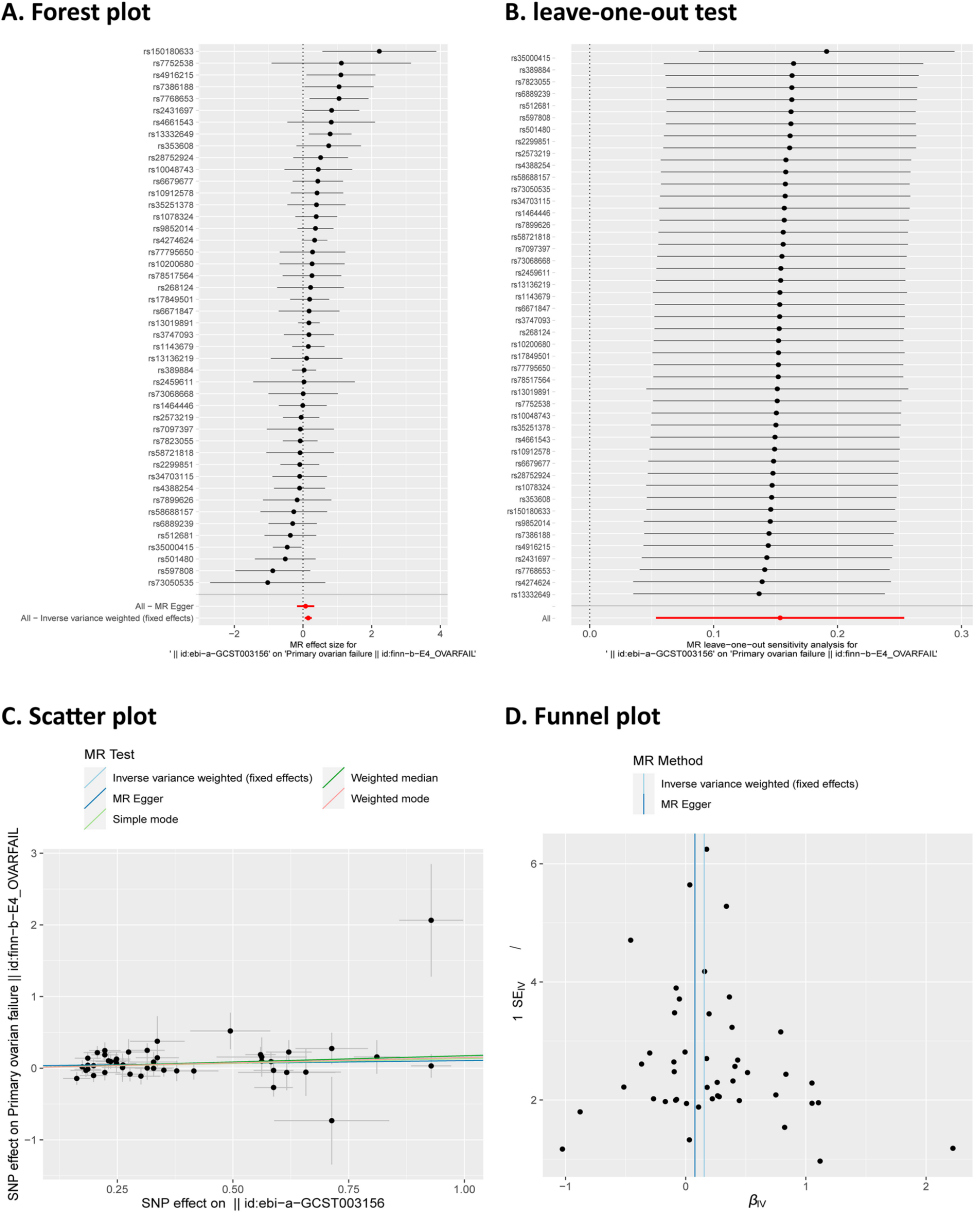
(**A**) Forest plot of the association between SLE and primary ovarian failure; (**B**) Sensitive analysis of leave-one-out test; (**C**) Scatter plot of the association between SLE and primary ovarian failure; (**D**) Sensitive analysis of funnel plot.

The results of the causal estimate between SLE and the risk of POF were shown in Fig. 3A as well as Fig. 4A,C. It should be noted that a significant positive association was found by using the main analytical method (IVW: OR = 1.166, 95% CI 1.055–1.289, *P* = 0.003) and one of the secondery analytical methods (Weighted median: OR = 1.190, 95% CI 1.018–1.391, *P* = 0.029). No significant association was seen using MR Egger, Weighted mode, and Simple mode.

For the heterogeneity test,the Cochrane Q statistics were demonstrated in Table 1. Since the *P*-value was greater than 0.05, no heterogeneity between SNPs was indicated. In Fig. 4 were other sensitivity analyses. The leave-one-out analysis revealed that the potential causal association between SLE and the risk of POF was not dominated by a single SNP (Fig. 4B). Moreover, the results of the MR Egger intercept test (Table 1) and funnel plot (Fig. 4D) showed no evidence of horizontal pleiotropy.

**Table 1:** Results from Cochrane Q test and MR Egger intercept test.

Disease	Cochrane Q test			MR-Egger intercept test		
	Q	Q_df	*P*-value	Intercept	SE	*P*-value
Primary ovarian failure	56.370	45	0.119	0.077	0.127	0.549
Age at menarche	16.778	20	0.667	− 0.010	0.010	0.344
Age at natural menopause	123.757	20	< 0.05	-0.060	0.091	0.516
Age at first live birth	52.275	45	0.212	0.005	0.005	0.332

### Causal association of SLE with other reproductive traits

The causal relationship between SLE and other reproductive traits (including age at menarche, age at menopause, and age at first live birth) were evaluated and forest plots, as well as a scatter plot, were demonstrated in Fig. 3 and Supplementary Figure S1–3. Though no significant association was found between SLE and age at menarche or menopause, SLE was correlated positively with age at first live birthaccording to the IVM method (OR = 1.006, 95% CI 1.002–1.010, *P* = 0.007).

After a test for Cochrane Q statistics (Table 1), only SNPs of age at menopause showed possible heterogeneity with a *P*-value lower than 0.05. No evidence of horizontal pleiotropy was found by the MR Egger intercept test (Table 1) or funnel plot (Supplementary Figure S1–3).The results from the leave-one-out analysis indicated no dominant SNP in the relationship between SLE and the three reproductive traits (Supplementary Figures S1–3).

## Discussion

In the present study, we conducted a two-sample MR study to investigate the causal relationship between SLE and the risk of POF as well as other female reproductive traits for the first time. The results supported a potential genetical causality between SLE and an increased risk of POF. Moreover, SLE might also be causally associated with delayed age at first live birth, with no evidence of a causal association between SLE and age at menopause or menarche. The sensitivity analysis demonstrated the absence of horizontal pleiotropy, suggesting the robustness of the effect estimates.

Previous observational studies have already indicated a possible association between SLE and POF. The epidemiological correlation, however, is partly due to the use of treatment medication (like CYC) for SLE that might damage ovarian function, given that a study conducted by Medeiros et.al concluded that CYC dose and age were the most significant risk factors contributing to the higher frequency of ovarian failure in SLE patients^23^. After eliminating the confounding factor of drug use, the correlation between SLE and ovarian failure became controversial. What Mayorga et.al found suggested that the prevalence of POF in SLE patients was consistent with that in the general population after excluding SLE patients undergoing CYC treatment^6^. In contrast, Pasoto et.al showed that in SLE patients, disease activity correlated significantly with menstrual disturbances in the absence of current and previous alkylating therapy^5^. Similarly, after evaluating 94 SLE patients, Shabanova et.al supported that increased disease activity might contribute to menstrual cycle disorders with oligomenorrhea before treatment with CYC and high doses of glucocorticoids^24^. Moreover, the ovarian reserve, as estimated by AMH, was also suggested to be significantly lower in SLE patients than in age-matched healthy controls^7^. The results of epidemiological observational studies might interfere with a few confounders, such as drug use and disease activity, which have been mentioned before, and there is a lack of randomized control trial studies offering conclusive evidence about the association between SLE and POF or menstrual cycle disturbance. In this circumstance, our study, by using MR analysis based on genetic variants, provides evidence for the causality between SLE and POF from a genetic perspective and avoids the bias that can occur in observational study.

The underlying mechanism of how SLE contributes to POF has not been elucidated. According to existing evidence, symptoms of POF including decreased sex steroid levels, premature menopause, and resistant ovary syndrome might be mainly caused by autoimmune ovarian damage and the affected endocrine environment in SLE patients. For ovarian damage caused by humoral immunity, it should be noted that SLE patients were capable of producing an explosion of more than 100 autoimmune antibodies^25^. Among them, antiphospholipid antibody (aPL) was proven to be associated with a decreased level of AMH, which represents an affected ovarian reserve^26^. In addition, autoimmune antibodies directed against the ovary have been widely detected in POF patients^27^, indicating a possible association between autoimmune disease and POF. When it comes to cellular immunity, evidence also showed a consistent increase of CD4 + T cells of peripheral blood in SLE and POF patients, which suggested an inner correlation between the two diseases^27–29^. What is also important is the unbalanced endocrine environment in SLE patients. The normal function of the hypothalamic-pituitary-ovarian (HPO) axis was known to be significant in maintaining female fertility and a regular menstrual cycle. Studies have pointed out that compared to healthy controls, SLE patients produced a higher level of follicle stimulation hormone (FSH) and a lower level of progesterone as well as luteinizing hormone (LH) regardless of CYC treatment, indicating dysfunction of the HPO axis and more specifically, of the ovarian response to gonadotropins in these patients^30,31^. Additionally, a significantly reduced concentration of total and free testosterone, which could be regarded as natural immunosuppressors in the humoral immunity, has also been found in both treated and untreated female lupus patients in comparison with the healthy controls^32,33^. In summary, although concrete evidence showing the underlying mechanisms is still urgently needed, it is reasonable to believe that both the injury of the ovary generated by autoimmunity and the chaos of the endocrine environment in SLE patients might contribute to POF, based on existing studies.

In our study, three other reproductive traits have been selected to further investigate the association between SLE and female reproduction. There was an absence of evidence showing that SLE was causally related to the age at menarche or age at menopause. This finding was consistent with that of Alpizar-Rodriguez et al. who, after analyzing 961 SLE patients, concluded that the mean age at natural menopause in SLE patients (50 years old) was similar to that in the general population^34^. For other reproductive traits, our study has offered clues to the potential causal association between SLE and age at first live birth. Previous studies have already pointed out that SLE patients might have a higher frequency of adverse fetal outcomes. Poh et.al, for instance, after evaluating a retrospective cohort study of 75 SLE pregnancies, found that adverse fetal outcomes including miscarriage, intrauterine fetal death, preterm delivery, and so on were in half of the pregnancies (53.3%), although the majority of the patients had quiescent SLE disease activity during pregnancy^35^. On the other hand, it should not be neglected that SLE might result in body image disturbance and cause sexual problems according to a Chinese study, which might also lead to delays in having children^36^. Consistent with the existing evidence, our study has indicated that SLE might contribute to adverse female fertility from a genetic perspective.

There are several strengths in our present study. First and foremost, as mentioned before, the design of MR can demonstrate or verify a causal relationship between exposure and outcomes in the absence of randomized clinical trials. By conducting a two-sample MR study and further sensitivity analysis, we demonstrated causality between SLE and the risk of POF as well as between SLE and delayed age at live birth, which avoided the confounders commonly found in observational studies. Second, we have utilized genetic data from large and well-powered GWASs of SLE, POF, and other reproductive traits. Moreover, we have adjusted a potential confounder BMI during the process of MR, which might influence SLE and reproductive traits at the same time^32,37^.

Several limitations still exist in the present study. First, to address the bias due to ethnic heterogeneity, our study limited the ethnicity to European ancestry, which, however, might also restrict application of our findings to other ethnicities. Second, although we have adjusted a potential confounder (BMI), there might be other potential confounders that we did not recognize in the present study. Thus, further studies considering more possible confounders are needed. Third, due to limited information, it is difficult to investigate the individual overlap between the selected population of SLE and reproductive traits, which might introduce weak instrument bias in the process of analysis^38^. Nevertheless, the test of *F*-statistics in the present study supported an absence of this kind of bias and there was no cohort overlap according to the information provided by these GWASs (Table S6), making our results more reliable. Moreover, it should be noted that there is a relatively small case number of POF GWAS (254 cases), and this number is also unbalanced compared to the number of controls (118,228 controls), which might be a potential limitation of our study. Therefore, to support out conclusions, larger and more powerful GWASs for POF are needed in the future.

## Conclusion

By performing a two-sample MR analysis, our study provided evidence of a causal correlation between SLE and a greater risk of POF. In addition, SLE might causally contribute to an increase in age at first live birth. Overall, our findings suggested an impact of SLE on female reproduction from a genetic perspective, providing insight into the basic disease mechanisms of SLE. And based on our research, the importance of early clinical care, comprehensive fertility assessment, and fertility preservation should be highlighted in SLE patients. In the future, additional studies investigating the underlying mechanisms concerning how SLE affects female reproduction are needed.

### Supplementary Information

Supplementary Figures.

Supplementary Tables.

## Data Availability

Data are available upon reasonable request by any qualified researchers who engage in rigorous, independent scientific research, and will be provided following review and approval of a research proposal and Statistical Analysis Plan (SAP) and execution of a Data Sharing Agreement (DSA). X.W. is responsible for data requestion. All data relevant to the study are included in the article.
