# Supplementary material for: The genetic relationship between systemic lupus erythematosus and risk of primary ovarian failure from a mendelian randomization study
Source: Sci Rep. 2024 Apr 24;14:9413. doi: 10.1038/s41598-024-59726-9 (PMC11043424; doi:10.1038/s41598-024-59726-9)

**Supplementary Figure S1.** A. Forest plot of the association between SLE and age at menarche; B. Sensitive analysis of leave-one-out test; C. Scatter plot of the association between SLE and age at menarche; D. Sensitive analysis of funnel plot.


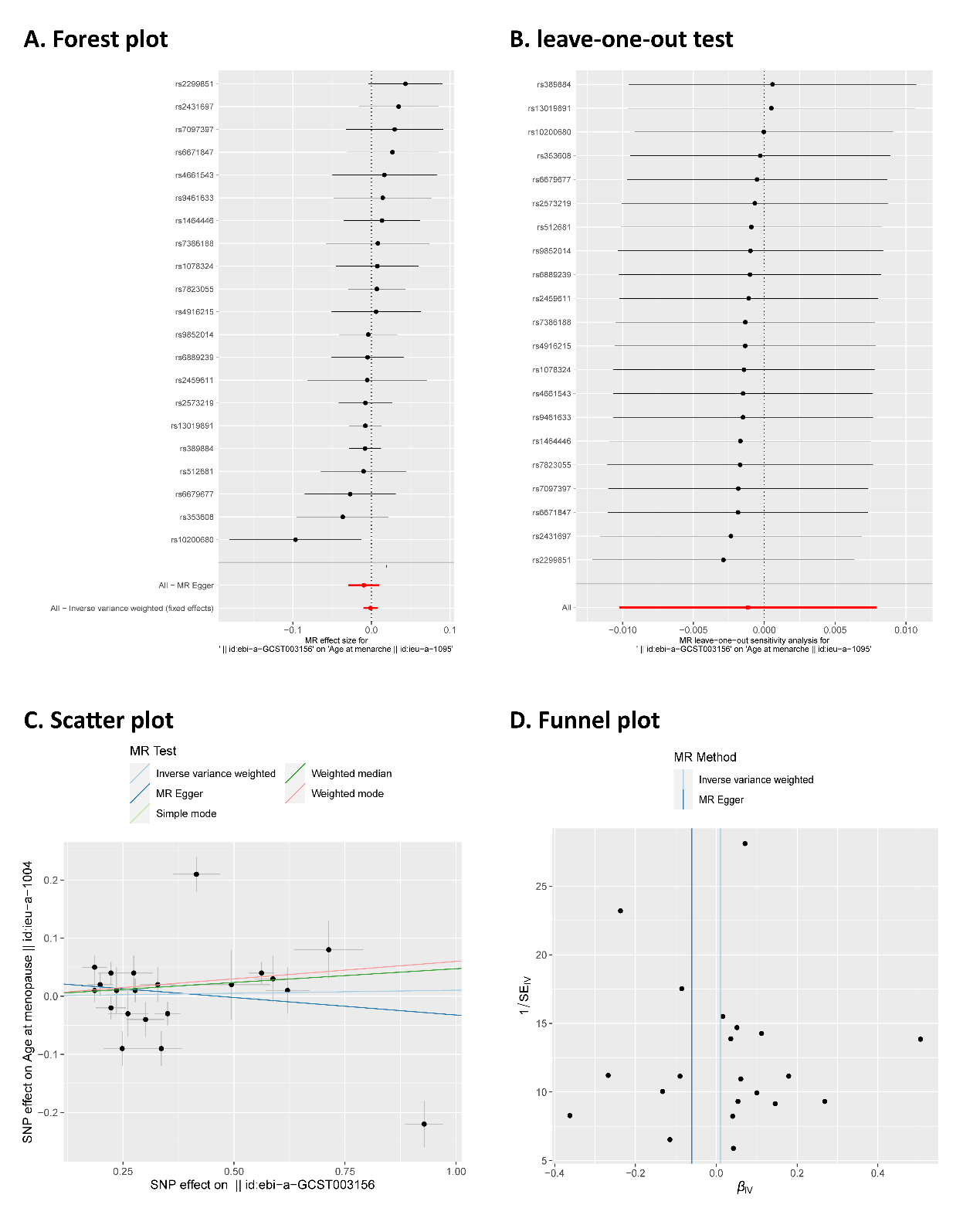


**Supplementary Figure S2.** A. Forest plot of the association between SLE and age at natural menopause; B. Sensitive analysis of leave-one-out test; C. Scatter plot of the association between SLE and age at natural menopause; D. Sensitive analysis of funnel plot.


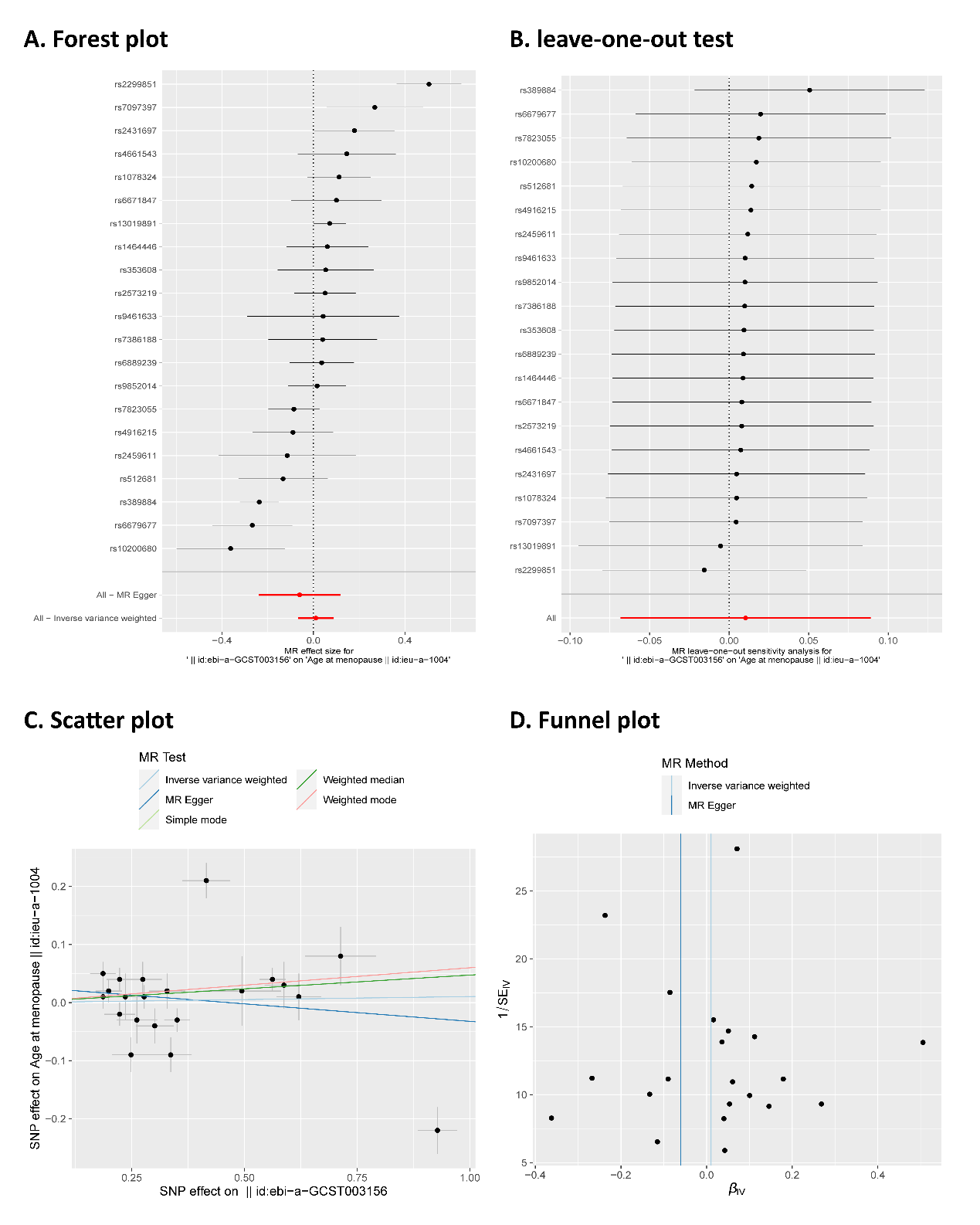


**Supplementary Figure S3.** A. Forest plot of the association between SLE and age at first live birth; B. Sensitive analysis of leave-one-out test; C. Scatter plot of the association between SLE and age at first live birth; D. Sensitive analysis of funnel plot.


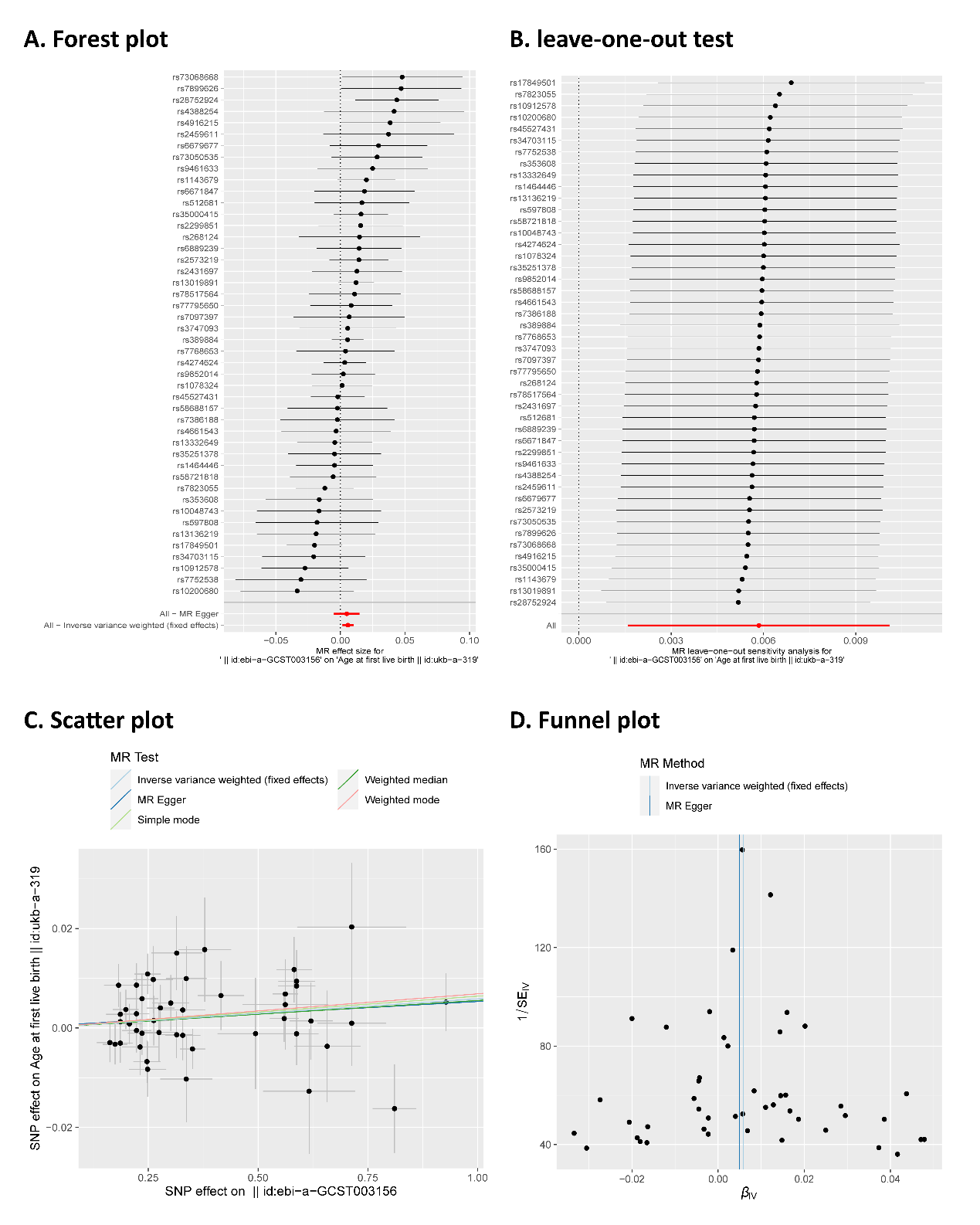

Supplement: Supplementary file 1 — Supplementary Figures. [file 41598_2024_59726_MOESM1_ESM.docx]
