# Supplementary material for: The genetic relationship between systemic lupus erythematosus and risk of primary ovarian failure from a mendelian randomization study
Source: Sci Rep. 2024 Apr 24;14:9413. doi: 10.1038/s41598-024-59726-9 (PMC11043424; doi:10.1038/s41598-024-59726-9)
Supplement: Supplementary file 2 — Supplementary Tables. [file 41598_2024_59726_MOESM2_ESM.docx]

**Supplementary Table S1.** Information of summary statistics of female reproductive traits.

| Traits | GWAS id of IEU | Ncase | Ncontrol | Source | Ancestry | Year |
| --- | --- | --- | --- | --- | --- | --- |
| Primary ovarian failure | finn-b-E4_OVARFAIL | 254 | 118,482 | FinnGen | European | 2021 |
| Age at menarche | ieu-a-1095 | 182,416 | | Reprogen | European | 2014 |
| Age at natural menopause | ieu-a-1004 | 69,360 | | Reprogen | European | 2015 |
| Age at first live birth | ukb-a-319 | 123,846 | | Neal Lab (UK biobank) | European | 2017 |

Note: Primary ovarian failure (POF) was defined by the International Statistical Classification of Diseases and Related Health Problems 10th Revision (ICD-10-E28.3). The diagnosis of POF is based on age less than forty, amenorrhea for four months or more, and two elevated serum follicle-stimulating hormone (FSH) (>40mIU/ml) measurements at one-month intervals.

**Supplementary Table S2.** Information of selected SNPs in the analysis of the association between systemic lupus erythematosus and premature ovarian failure.

| SNP | Chr | Pos | Effect_allele | Other_allele | Eaf | Beta | Se | F_statistics |
| --- | --- | --- | --- | --- | --- | --- | --- | --- |
| rs10048743 | 2 | 213890232 | T | G | 0.82 | -0.23 | 0.04 | 231.05 |
| rs10200680 | 2 | 223961877 | T | C | 0.17 | -0.25 | 0.04 | 251.73 |
| rs1078324 | 5 | 149202268 | A | C | 0.04 | -0.71 | 0.08 | 602.19 |
| rs10912578 | 1 | 173251856 | G | A | 0.68 | -0.25 | 0.03 | 386.46 |
| rs1143679 | 16 | 31276811 | A | G | 0.12 | 0.58 | 0.04 | 1068.44 |
| rs13019891 | 2 | 113829869 | T | G | 0.46 | -0.56 | 0.03 | 2657.15 |
| rs13136219 | 4 | 102743687 | T | C | 0.37 | -0.17 | 0.03 | 204.38 |
| rs13332649 | 16 | 85966683 | G | A | 0.28 | -0.31 | 0.04 | 589.19 |
| rs1464446 | 3 | 146601295 | T | G | 0.18 | -0.33 | 0.04 | 463.30 |
| rs150180633 | 6 | 31010047 | T | C | 0.00 | 0.93 | 0.07 | 96.09 |
| rs17849501 | 1 | 183542323 | T | C | 0.04 | 0.81 | 0.05 | 728.38 |
| rs2299851 | 6 | 31718602 | A | G | 0.17 | -0.42 | 0.05 | 733.55 |
| rs2431697 | 5 | 159879978 | C | T | 0.39 | -0.22 | 0.03 | 345.56 |
| rs2459611 | 2 | 191939187 | T | C | 0.95 | 0.26 | 0.05 | 99.03 |
| rs2573219 | 2 | 233288667 | C | A | 0.09 | 0.59 | 0.04 | 811.22 |
| rs268124 | 2 | 65654364 | T | C | 0.62 | 0.19 | 0.03 | 236.06 |
| rs28752924 | 6 | 31303922 | C | T | 0.44 | -0.25 | 0.03 | 448.61 |
| rs34703115 | 2 | 40282854 | C | T | 0.03 | -0.62 | 0.10 | 354.39 |
| rs35000415 | 7 | 128585616 | T | C | 0.15 | 0.59 | 0.04 | 1384.08 |
| rs35251378 | 19 | 10459969 | A | G | 0.26 | -0.24 | 0.03 | 313.51 |
| rs353608 | 11 | 35101738 | G | A | 0.53 | 0.19 | 0.03 | 250.83 |
| rs3747093 | 22 | 21984379 | A | G | 0.31 | 0.26 | 0.03 | 435.67 |
| rs389884 | 6 | 31940897 | G | A | 0.09 | 0.93 | 0.04 | 2396.41 |
| rs4274624 | 2 | 191958656 | T | C | 0.77 | -0.56 | 0.03 | 1785.98 |
| rs4388254 | 5 | 133428601 | T | C | 0.11 | 0.38 | 0.06 | 419.65 |
| rs4661543 | 1 | 15229101 | G | T | 0.93 | 0.27 | 0.04 | 135.42 |
| rs4916215 | 1 | 173314540 | T | C | 0.82 | 0.22 | 0.03 | 215.71 |
| rs501480 | 6 | 33563946 | C | T | 0.50 | 0.20 | 0.03 | 287.73 |
| rs512681 | 2 | 119450163 | A | C | 0.19 | -0.30 | 0.04 | 401.16 |
| rs58688157 | 11 | 625085 | G | A | 0.21 | -0.22 | 0.03 | 236.57 |
| rs58721818 | 6 | 138243739 | T | C | 0.02 | 0.66 | 0.08 | 223.02 |
| rs597808 | 12 | 111973358 | G | A | 0.58 | -0.16 | 0.03 | 185.38 |
| rs6671847 | 1 | 161478810 | A | G | 0.45 | 0.20 | 0.03 | 285.23 |
| rs6679677 | 1 | 114303808 | A | C | 0.15 | 0.34 | 0.05 | 419.29 |
| rs6889239 | 5 | 150457771 | C | T | 0.29 | 0.28 | 0.03 | 464.89 |
| rs7097397 | 10 | 50025396 | A | G | 0.37 | -0.19 | 0.03 | 234.68 |
| rs73050535 | 12 | 5012503 | T | C | 0.00 | -0.71 | 0.12 | 71.98 |
| rs73068668 | 19 | 55763262 | A | G | 0.08 | -0.31 | 0.06 | 218.54 |
| rs7386188 | 8 | 50609940 | G | T | 0.97 | 0.49 | 0.09 | 212.53 |
| rs7752538 | 6 | 34674502 | A | G | 0.02 | 0.34 | 0.06 | 51.73 |
| rs7768653 | 6 | 106574794 | T | C | 0.53 | -0.21 | 0.03 | 310.85 |
| rs77795650 | 6 | 32452083 | A | G | 0.03 | -0.56 | 0.10 | 259.52 |
| rs7823055 | 8 | 55511676 | T | G | 0.57 | -0.35 | 0.03 | 916.24 |
| rs78517564 | 1 | 183442324 | C | A | 0.11 | -0.33 | 0.05 | 303.93 |
| rs7899626 | 10 | 63825561 | T | C | 0.39 | 0.18 | 0.03 | 229.79 |
| rs9852014 | 3 | 129084581 | G | A | 0.08 | 0.62 | 0.05 | 855.77 |

**Supplementary Table S3.** Information of selected SNPs in the analysis of the association between systemic lupus erythematosus and age at menarche.

| SNP | Chr | Pos | Effect_allele | Other_allele | Eaf | Beta | Se | F_statistics |
| --- | --- | --- | --- | --- | --- | --- | --- | --- |
| rs10200680 | 2 | 223961877 | T | C | 0.05 | -0.25 | 0.04 | 84.16 |
| rs1078324 | 5 | 149202268 | A | C | 0.03 | -0.71 | 0.08 | 435.40 |
| rs13019891 | 2 | 113829869 | T | G | 0.45 | -0.56 | 0.03 | 2645.04 |
| rs1464446 | 3 | 146601295 | T | G | 0.27 | -0.33 | 0.04 | 633.84 |
| rs2299851 | 6 | 31718602 | A | G | 0.12 | -0.42 | 0.05 | 539.89 |
| rs2431697 | 5 | 159879978 | C | T | 0.47 | -0.22 | 0.03 | 362.90 |
| rs2459611 | 2 | 191939187 | T | C | 0.91 | 0.26 | 0.05 | 161.44 |
| rs2573219 | 2 | 233288667 | C | A | 0.13 | 0.59 | 0.04 | 1209.41 |
| rs353608 | 11 | 35101738 | G | A | 0.57 | 0.19 | 0.03 | 247.00 |
| rs389884 | 6 | 31940897 | G | A | 0.09 | 0.93 | 0.04 | 2344.17 |
| rs4661543 | 1 | 15229101 | G | T | 0.88 | 0.27 | 0.04 | 230.59 |
| rs4916215 | 1 | 173314540 | T | C | 0.73 | 0.22 | 0.03 | 285.63 |
| rs512681 | 2 | 119450163 | A | C | 0.15 | -0.30 | 0.04 | 337.63 |
| rs6671847 | 1 | 161478810 | A | G | 0.49 | 0.20 | 0.03 | 287.62 |
| rs6679677 | 1 | 114303808 | A | C | 0.11 | 0.34 | 0.05 | 323.41 |
| rs6889239 | 5 | 150457771 | C | T | 0.23 | 0.28 | 0.03 | 400.42 |
| rs7097397 | 10 | 50025396 | A | G | 0.38 | -0.19 | 0.03 | 237.27 |
| rs7386188 | 8 | 50609940 | G | T | 0.92 | 0.49 | 0.09 | 532.22 |
| rs7823055 | 8 | 55511676 | T | G | 0.57 | -0.35 | 0.03 | 915.04 |
| rs9461633 | 6 | 30761168 | G | A | 0.15 | -0.24 | 0.04 | 205.04 |
| rs9852014 | 3 | 129084581 | G | A | 0.06 | 0.62 | 0.05 | 647.87 |

**Supplementary Table S4.** Information of selected SNPs in the analysis of the association between systemic lupus erythematosus and age at natural menopause.

| SNP | Chr | Pos | Effect_allele | Other_allele | Eaf | Beta | Se | F_statistics |
| --- | --- | --- | --- | --- | --- | --- | --- | --- |
| rs10200680 | 2 | 223961877 | T | C | 0.05 | -0.25 | 0.04 | 84.16 |
| rs1078324 | 5 | 149202268 | A | C | 0.03 | -0.71 | 0.08 | 435.40 |
| rs13019891 | 2 | 113829869 | T | G | 0.45 | -0.56 | 0.03 | 2645.04 |
| rs1464446 | 3 | 146601295 | T | G | 0.27 | -0.33 | 0.04 | 633.84 |
| rs2299851 | 6 | 31718602 | A | G | 0.12 | -0.42 | 0.05 | 539.89 |
| rs2431697 | 5 | 159879978 | C | T | 0.47 | -0.22 | 0.03 | 362.90 |
| rs2459611 | 2 | 191939187 | T | C | 0.91 | 0.26 | 0.05 | 161.44 |
| rs2573219 | 2 | 233288667 | C | A | 0.13 | 0.59 | 0.04 | 1209.41 |
| rs353608 | 11 | 35101738 | G | A | 0.57 | 0.19 | 0.03 | 247.00 |
| rs389884 | 6 | 31940897 | G | A | 0.09 | 0.93 | 0.04 | 2344.17 |
| rs4661543 | 1 | 15229101 | G | T | 0.87 | 0.27 | 0.04 | 247.25 |
| rs4916215 | 1 | 173314540 | T | C | 0.73 | 0.22 | 0.03 | 285.63 |
| rs512681 | 2 | 119450163 | A | C | 0.15 | -0.30 | 0.04 | 337.63 |
| rs6671847 | 1 | 161478810 | A | G | 0.48 | 0.20 | 0.03 | 287.27 |
| rs6679677 | 1 | 114303808 | A | C | 0.10 | 0.34 | 0.05 | 296.77 |
| rs6889239 | 5 | 150457771 | C | T | 0.23 | 0.28 | 0.03 | 400.42 |
| rs7097397 | 10 | 50025396 | A | G | 0.38 | -0.19 | 0.03 | 237.27 |
| rs7386188 | 8 | 50609940 | G | T | 0.92 | 0.49 | 0.09 | 532.22 |
| rs7823055 | 8 | 55511676 | T | G | 0.57 | -0.35 | 0.03 | 915.04 |
| rs9461633 | 6 | 30761168 | G | A | 0.15 | -0.24 | 0.04 | 205.04 |
| rs9852014 | 3 | 129084581 | G | A | 0.06 | 0.62 | 0.05 | 647.87 |

**Supplementary Table S5.** Information of selected SNPs in the analysis of the association between systemic lupus erythematosus and age at first live birth.

| SNP | Chr | Pos | Effect_allele | Other_allele | Eaf | Beta | Se | F_statistics |
| --- | --- | --- | --- | --- | --- | --- | --- | --- |
| rs10048743 | 2 | 213890232 | T | G | 0.86 | -0.23 | 0.04 | 186.11 |
| rs10200680 | 2 | 223961877 | T | C | 0.15 | -0.25 | 0.04 | 224.90 |
| rs1078324 | 5 | 149202268 | A | C | 0.06 | -0.71 | 0.08 | 818.58 |
| rs10912578 | 1 | 173251856 | G | A | 0.67 | -0.25 | 0.03 | 392.53 |
| rs1143679 | 16 | 31276811 | A | G | 0.10 | 0.58 | 0.04 | 935.78 |
| rs13019891 | 2 | 113829869 | T | G | 0.46 | -0.56 | 0.03 | 2655.32 |
| rs13136219 | 4 | 102743687 | T | C | 0.37 | -0.17 | 0.03 | 206.10 |
| rs13332649 | 16 | 85966683 | G | A | 0.23 | -0.31 | 0.04 | 513.77 |
| rs1464446 | 3 | 146601295 | T | G | 0.19 | -0.33 | 0.04 | 496.42 |
| rs17849501 | 1 | 183542323 | T | C | 0.05 | 0.81 | 0.05 | 989.07 |
| rs2299851 | 6 | 31718602 | A | G | 0.09 | -0.42 | 0.05 | 410.60 |
| rs2431697 | 5 | 159879978 | C | T | 0.43 | -0.22 | 0.03 | 357.68 |
| rs2459611 | 2 | 191939187 | T | C | 0.90 | 0.26 | 0.05 | 172.21 |
| rs2573219 | 2 | 233288667 | C | A | 0.09 | 0.59 | 0.04 | 863.63 |
| rs268124 | 2 | 65654364 | T | C | 0.73 | 0.19 | 0.03 | 196.47 |
| rs28752924 | 6 | 31303922 | C | T | 0.46 | -0.25 | 0.03 | 451.11 |
| rs34703115 | 2 | 40282854 | C | T | 0.02 | -0.62 | 0.10 | 268.44 |
| rs35000415 | 7 | 128585616 | T | C | 0.11 | 0.59 | 0.04 | 1045.70 |
| rs35251378 | 19 | 10459969 | A | G | 0.29 | -0.24 | 0.03 | 336.05 |
| rs353608 | 11 | 35101738 | G | A | 0.51 | 0.19 | 0.03 | 251.94 |
| rs3747093 | 22 | 21984379 | A | G | 0.20 | 0.26 | 0.03 | 316.63 |
| rs389884 | 6 | 31940897 | G | A | 0.13 | 0.93 | 0.04 | 3536.54 |
| rs4274624 | 2 | 191958656 | T | C | 0.77 | -0.56 | 0.03 | 1751.71 |
| rs4388254 | 5 | 133428601 | T | C | 0.04 | 0.38 | 0.06 | 144.98 |
| rs45527431 | 6 | 26599509 | G | A | 0.11 | 0.59 | 0.05 | 1046.02 |
| rs4661543 | 1 | 15229101 | G | T | 0.87 | 0.27 | 0.04 | 240.52 |
| rs4916215 | 1 | 173314540 | T | C | 0.73 | 0.22 | 0.03 | 285.95 |
| rs512681 | 2 | 119450163 | A | C | 0.15 | -0.30 | 0.04 | 334.53 |
| rs58688157 | 11 | 625085 | G | A | 0.28 | -0.22 | 0.03 | 291.78 |
| rs58721818 | 6 | 138243739 | T | C | 0.03 | 0.66 | 0.08 | 395.91 |
| rs597808 | 12 | 111973358 | G | A | 0.52 | -0.16 | 0.03 | 190.71 |
| rs6671847 | 1 | 161478810 | A | G | 0.51 | 0.20 | 0.03 | 287.58 |
| rs6679677 | 1 | 114303808 | A | C | 0.10 | 0.34 | 0.05 | 303.68 |
| rs6889239 | 5 | 150457771 | C | T | 0.23 | 0.28 | 0.03 | 406.29 |
| rs7097397 | 10 | 50025396 | A | G | 0.37 | -0.19 | 0.03 | 233.49 |
| rs73050535 | 12 | 5012503 | T | C | 0.02 | -0.71 | 0.12 | 354.75 |
| rs73068668 | 19 | 55763262 | A | G | 0.08 | -0.31 | 0.06 | 200.95 |
| rs7386188 | 8 | 50609940 | G | T | 0.97 | 0.49 | 0.09 | 217.54 |
| rs7752538 | 6 | 34674502 | A | G | 0.05 | 0.34 | 0.06 | 164.50 |
| rs7768653 | 6 | 106574794 | T | C | 0.60 | -0.21 | 0.03 | 300.01 |
| rs77795650 | 6 | 32452083 | A | G | 0.06 | -0.56 | 0.10 | 568.40 |
| rs7823055 | 8 | 55511676 | T | G | 0.57 | -0.35 | 0.03 | 916.10 |
| rs78517564 | 1 | 183442324 | C | A | 0.12 | -0.33 | 0.05 | 343.38 |
| rs7899626 | 10 | 63825561 | T | C | 0.30 | 0.18 | 0.03 | 201.80 |
| rs9461633 | 6 | 30761168 | G | A | 0.18 | -0.24 | 0.04 | 236.67 |
| rs9852014 | 3 | 129084581 | G | A | 0.07 | 0.62 | 0.05 | 756.10 |

**Supplementary Table S6. Information of cohorts included in the GWAS studies.**

| Traits | Description | Number of cases | Number of controls |
| --- | --- | --- | --- |
| Systemic Lupus Erythematosus^1^ | Canadian samples | 823 | 0 |
|  | Spanish samples, coordinated by a center in Barcelona | 492 | 271 |
|  | Dutch and Polish samples, coordinated by a center in the Netherlands | 256 | 0 |
|  | Spanish samples, coordinated by a center in Granada | 452 | 99 |
|  | German samples | 194 | 0 |
|  | Turkish samples | 77 | 128 |
|  | American samples | 188 | 0 |
|  | UK samples, provided by the MRC and 1958 British Birth Cohort | 87 | 231 |
|  | Samples provided by the BIOLUPUS network (northern European) | 407 | 16 |
|  | Samples provided by the BIOLUPUS and GENLES networks (southern European) | 704 | 541 |
|  | UK samples | 1,266 | 0 |
|  | University of Michigan Health and Retirement Study (HRS) | 0 | 5,727 |
|  | North Americans of European descent sample | 1,165 | 2,107 |
| Primary ovarian failure | FinnGen Cohort | 254 | 118,482 |
| Age at menarche | UK samples from UK biobank | 182,416 | |
| Age at natural menopause | UK samples from UK biobank | 69,360 | |
| Age at first live birth | UK samples from UK biobank | 123,846 | |
| 1: All samples before quality control analyses were listed here. | | | |
